# The association between IGF1 gene rs1520220 polymorphism and cancer susceptibility: a meta-analysis based on 12,884 cases and 58,304 controls

**DOI:** 10.1186/s12199-018-0727-y

**Published:** 2018-08-16

**Authors:** Gui-Ping Xu, Wei-Xian Chen, Wen-Yue Xie, Li-Fang Wu

**Affiliations:** 1 Transfusion Department, The Second Affiliated Hospital of Chongqing Medical University, Chongqing, China; 2 Department of Laboratory Medicine, The Second Affiliated Hospital of Chongqing Medical University, Chongqing, 400010 China; 3 Department of Oncology, The Second Affiliated Hospital of Chongqing Medical University, Chongqing, China

**Keywords:** rs1520220, IGF1, Polymorphism, Meta-analysis, Cancer

## Abstract

**Background:**

The rs1520220 polymorphism in the insulin-like growth factor 1 (*IGF1*) gene has been reported to affect cancer susceptibly in several studies. However, the results of the relevant studies are inconsistent. We conduct a current meta-analysis to investigate the association between rs1520220 and cancer susceptibly.

**Methods:**

Three databases (PubMed, Embase, and Web of Science) were searched for studies regarding the relationship between rs1520220 and cancer susceptibly. Odds ratios (ORs) and the related 95% confidence intervals (CIs) were employed to assess the strength of the associations. A stratified analysis was performed according to cancer type, ethnicity, and quality score, and when results were obtained from no fewer than two studies, these results were pooled.

**Results:**

There was no positive association between rs1520220 and overall cancer risk. However, the analysis stratified by ethnicity revealed that rs1520220 significantly increased cancer susceptibility in Asian populations (allele model OR = 1.10, 95%Cl = 1.00–1.21, *p* = 0.040; homozygote model OR = 1.22, 95%Cl = 1.01–1.47, *p* = 0.040; dominant model OR = 1.19, 95%Cl = 1.01–1.39, *p* = 0.033). No significantly association was detected in Caucasian populations. The analysis stratified by cancer type suggested that rs1520220 was not associated with susceptibility to breast cancer.

**Conclusions:**

The results of our meta-analysis demonstrate that the role of *IGF1* rs1520220 in cancer susceptibility varies by ethnicity and cancer type and that rs1520220 increases cancer susceptibility in Asian populations.

**Electronic supplementary material:**

The online version of this article (10.1186/s12199-018-0727-y) contains supplementary material, which is available to authorized users.

## Background

The occurrence of cancer depends on both genetic and environmental factors [1, 2]. Relevant environmental factors include pollution, tobacco and alcohol intake, overweight, and infection [3]. Studies based on twins have found that genetic factors are also an important risk factor for cancer [2, 4]. Recently, the role of SNPs in the occurrence and development of cancer has attracted increasing attention [5]. The SNPs that are associated with cancer risk may act as biomarkers for cancer diagnosis [6, 7].

IGF1 is a growth factor that involves in many important biological and pathological processes [8, 9]. The important functions of IGF1 are promoting cell proliferation and inhibiting apoptosis [10]. IGF1 has also been reported to be involved in cancer development [11]. Plasma IGF1 levels depend on many factors, such as BMI, but gene is also an important factor [12, 13]. Many studies have reported that several *IGF1* SNPs affect plasma IGF1 levels and thus influence the risk of cancer [14, 15].

rs1520220 is located in intron 3 of *IGF1* gene which might lead to alternative splicing and a subsequent change in protein function [16]. It has been reported that rs1520220 G to C substitution leads to increased plasma IGF1 level, increasing cancer risk as a result [13, 17]. However, the studies regarding the relationship between rs1520220 and cancer susceptibility are inconsistent [18–25]. For instance, Al-Zahrani et al. reported that rs1520220 increased susceptibility to breast cancer [18], but Li et al. suggested that rs1520220 was not related to susceptibility to breast cancer [25]. Considering the disagreement between these studies, we performed a meta-analysis of the associations between rs1520220 and cancer susceptibility to review these results and draw a more accurate conclusion.

## Methods

### Search strategy

We searched for relevant studies in three databases: PubMed, Embase, and Web of Science. The search conditions limited the language to English and the data of publication prior to February 28, 2018. The following keywords were used: “IGF1 or IGF-1 or insulin-like growth factor 1 or rs1520220,” “cancer or tumor or carcinoma,” and “SNP or polymorphism or variant or mutation.” We also checked the references of the identified articles to ensure that we obtained all potentially relevant studies.

### Inclusion and exclusion criteria

The inclusion criteria of this meta-analysis are as follows: studies must (1) concern the relationship between rs1520220 and cancer susceptibility, (2) be case-control or cohort study, and (3) contain sufficient genotyping data to allow for the pooling of the results (the GG, GC, and CC genotype frequencies in the case and control groups were provided directly or could be calculated from the provided data). The exclusion criteria are as follows: (1) when subjects of two studies overlap, the one containing fewer subjects was excluded, and (2) reviews and meta-analyses are excluded.

### Data extraction

The following information was extracted from the included studies by two authors independently: first author’s name, year of publication, country, cancer type, ethnicity, genotyping methods, control source, genotype distributions of cases and controls, and Hardy-Weinberg equilibrium (HWE) for controls. Disagreements were resolved via discussion.

### Quality score

We assessed the quality of the included studies based on the following five factors [26]: case source, control source, specimens used for determining genotypes, HWE in controls, and total sample size (Additional file 1: Table S1). A perfect score was 15.

### Statistical analysis

We estimated the strengths of the associations using pooled ORs with corresponding 95% CIs. Five genetic models are employed: the allele model (C vs. G), the homozygote model (CC vs. GG), the heterozygote model (GC vs. GG), the dominant model (CC + GC vs. GG), and the recessive model (CC vs. GC + GG). The heterogeneity was evaluated using a *Q* test and quantified by *I*^2^ [27]. When heterogeneity not exists (*P* > 0.1), the fixed-effects model was used [28]. Otherwise, the random-effects model was applied [29]. Hardy-Weinberg equilibrium (HWE) for controls was assessed using a chi-squared test. *P* values less than 0.05 were considered to indicate significant disequilibrium. Stratified analyses were conducted by ethnicity, cancer type, and quality score. Only results synthesized from no fewer than two studies are shown. Sensitivity analyses were performed via *metainf* command which investigates the influence of each individual study on the overall meta-analysis summary estimate by omitting each study in turn [30]. Publication bias was assessed using Begg’s test and Egger’s test [31, 32]. All statistical analyses were performed using the STATA software (Version 12.0; Stata Corporation, College Station, TX, USA).

## Results

### Characteristics of the studies

**Fig. 1:**
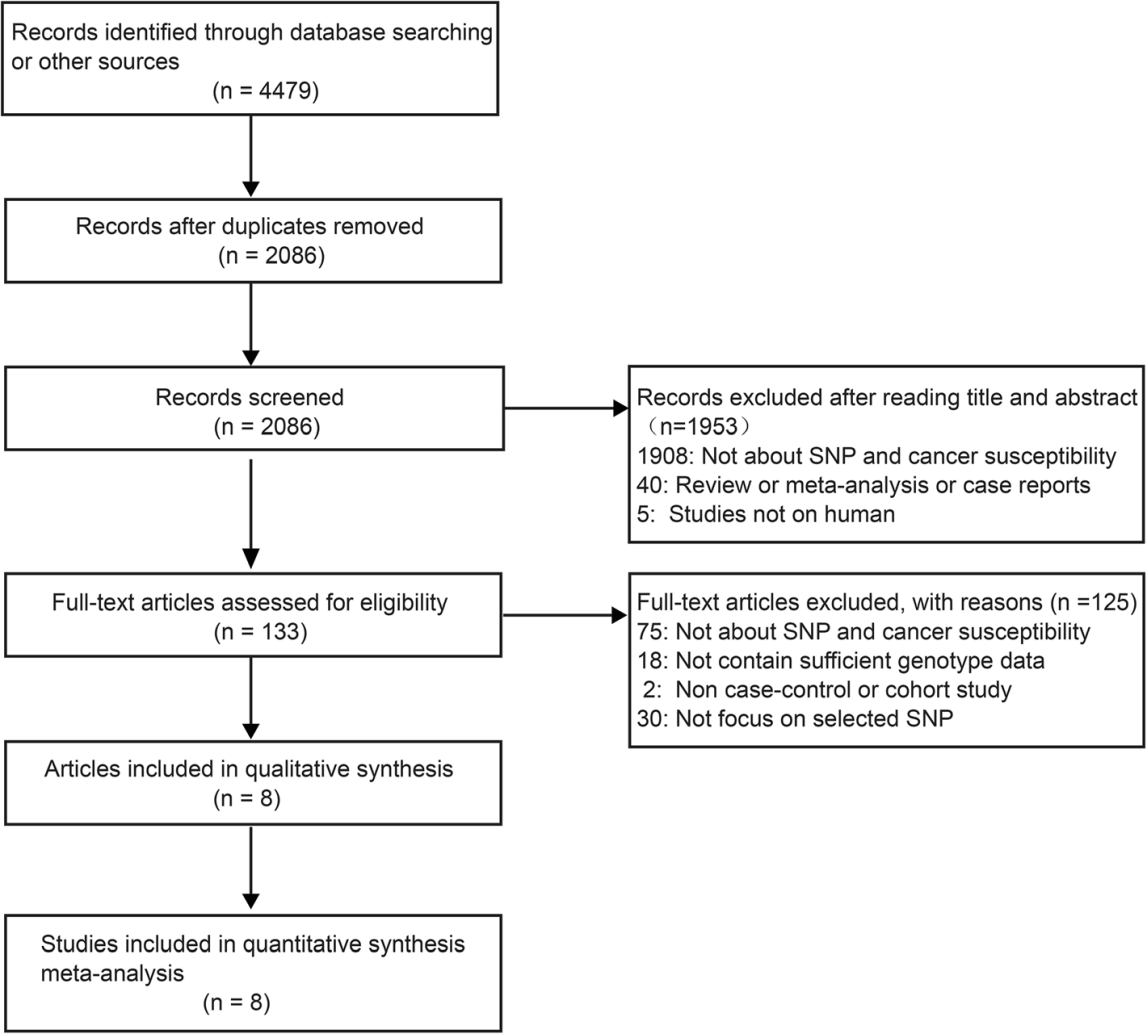
The flow diagram of included/excluded studies

We obtained 2086 relevant articles through database searching after removing duplicates. Then, by screening the titles and abstracts, we excluded 1953 articles, and 133 articles remained. We read the full texts of these 133 articles and ultimately identified eight articles that meet the inclusion criteria (Fig. 1), which involved 12,884 cases and 58,304 controls. The characteristics of the included studies are shown in Table 1. Among these eight studies, four were carried out in Asian populations, two were carried out in Caucasian populations, and two were carried out in mix populations. Four of them concerned breast cancer, and four concerned other cancers including testicular germ cell tumors (TGCT), stomach cancer, pancreatic cancer, and colorectal cancer. Seven of the included studies had quality scores of no less than 12. The distributions of the genotypes and allele frequencies in the cases and controls are shown in Table 2.

**Table 1:** Characteristics of the studies included in the meta-analysis

First author	Year	Country/region	Ethnicity	Cancer type	Genotyping method	Control source
AI-Zahrani [18]	2006	Europe	Caucasian	Breast cancer	TaqMan	PB
Chia [19]	2008	USA	Mix	TGCT	TaqMan	PB
Patel [20]	2008	USA or Europe	Mix	Breast cancer	TaqMan	PB
Ennishi [21]	2011	Japan	Asian	Stomach cancer	TaqMan	HB
Nakao [22]	2011	Japan	Asian	Pancreatic cancer	TaqMan	HB
Qian [23]	2011	China	Asian	Breast cancer	TaqMan	HB
Simons [24]	2015	Netherlands	Caucasian	Colorectal cancer	SEQUENOM® MassARRAY®	PB
Li [25]	2016	China	Asian	Breast cancer	TaqMan	PB

*TGCT* testicular germ cell tumors, *PB* population-based, *HB* hospital-based

**Table 2:** *IGF1* rs1520220 polymorphism genotype distribution and allele frequency in cases and controls

	Genotype (*N*)								Allele frequency (N)				HWE	Score
	Case				Control				Case		Control			
	Total	GG	GC	CC	Total	GG	GC	CC	**G**	**C**	**G**	**C**		
AI-Zahrani [18]	2036	1388	569	79	2194	1525	617	52	3345	727	3667	721	0.261	15
Chia [19]	568	378	169	21	698	452	209	37	925	211	1113	283	0.052	15
Patel [20]	6584	329	2207	4048	8424	440	2707	5277	2865	10,303	3587	13,261	< 0.001	12
Ennishi [21]	703	148	357	198	1462	361	728	373	653	753	1450	1474	0.877	12
Nakao [22]	176	33	97	46	1402	347	697	358	163	189	1391	1413	0.833	12
Qian [23]	403	71	189	143	403	78	193	132	331	475	349	457	0.620	11
Simons [24]	2274	85	691	1498	43,561	1384	12,689	29,488	861	3687	15,457	71,665	0.673	15
Li [25]	140	38	58	44	160	36	69	55	134	146	141	179	0.113	12

*HWE* Hardy-Weinberg equilibrium

### Meta-analysis

We investigate the role of rs1520220 polymorphisms in cancer susceptibility via pooled OR and 95%Cl. Only results synthesized from no fewer than two studies are shown. In the overall analysis, we did not find positive associations between rs1520220 and cancer susceptibility (Table 3).

**Table 3:** Meta-analysis of the association between *IGF1* rs1520220 polymorphism and cancer susceptibility

Subgroup	No.	C vs. G			CC vs. GG			GC vs. GG			CC + GC vs. GG			CC vs. GC + GG		
		OR (95%Cl)	*P*_OR_	*P*_*h*_	OR (95%Cl)	*P*_*OR*_	*P*_*h*_	OR (95%Cl)	*P*_OR_	*P*_*h*_	OR (95%Cl)	*P*_OR_	*P*_*h*_	OR (95%Cl)	*P*_OR_	*P*_*h*_
Overall	8	1.01 (0.95–1.08)^*^	0.715	0.026	1.08 (0.90–1.30)^*^	0.415	0.010	1.05 (0.97–1.13)	0.268	0.374	1.05 (0.97–1.13)	0.237	0.163	1.02 (0.91–1.13)^*^	0.773	0.026
Caucasian	2	1.00 (0.84–1.20)^*^	0.953	0.010	1.16 (0.58–2.31)^*^	0.675	0.001	0.98 (0.87–1.10)	0.743	0.330	0.97 (0.77–1.21)^*^	0.762	0.08	1.21 (0.68–2.15)^*^	0.524	0.002
Asian	4	*1.10 (1.00–1.21)*	*0.040*	0.455	*1.22 (1.01–1.47)*	*0.040*	0.426	1.17 (0.99–1.39)	0.066	0.382	*1.19 (1.01–1.39)*	*0.033*	0.324	1.10 (0.95–1.26)	0.215	0.762
Breast cancer	4	1.00 (0.95–1.05)	0.978	0.130	1.15 (0.87–1.51)^*^	0.320	0.053	1.04 (0.94–1.15)	0.425	0.713	1.05 (0.96–1.15)	0.289	0.703	1.11 (0.86–1.42)^*^	0.428	0.016
Quality score ≥ 12	7	1.01 (0.94–1.08)^*^	0.875	0.021	1.07 (0.87–1.31)^*^	0.542	0.006	1.04 (0.96–1.13)	0.292	0.275	1.04 (0.97–1.12)	0.283	0.112	1.01 (0.90–1.13)^*^	0.922	0.023

*OR* odds ratio, *95% CI* 95% confidence interval, *P*_OR_, pool *p* value; *P*_*h*_, *p* value of heterogeneity test

*Indicates that the OR, 95% Cl, and corresponding *P*_OR_ were calculated based on the random-effects model; otherwise, the fixed-effects model was used. Italic values are statistically significant (*P* < 0.05)

**Fig. 2:**
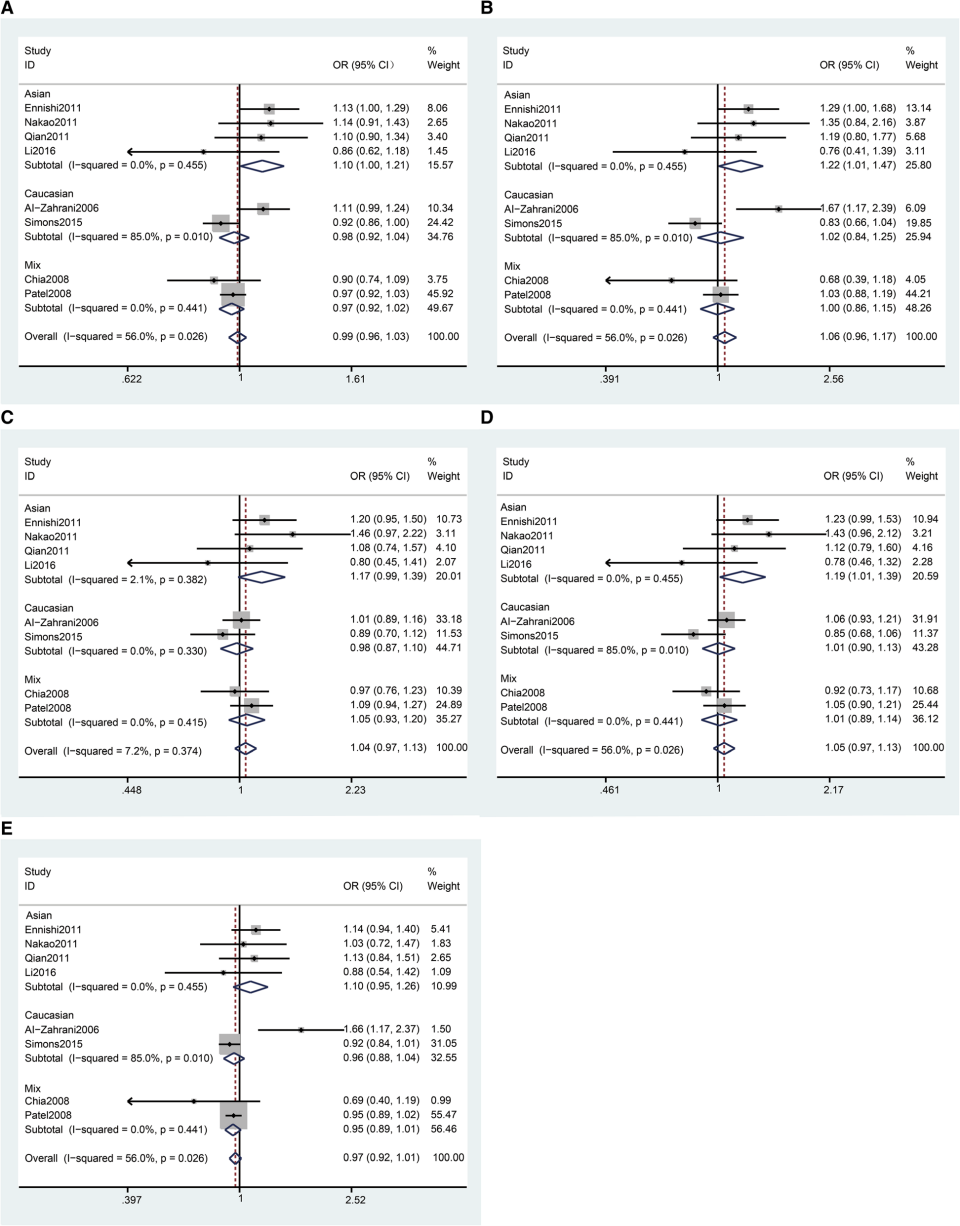
Stratification analyses by ethnicity between *IGF1* rs1520220 polymorphism and cancer susceptibility. **a** Allele model. **b** Homozygous model, **c** Heterozygous model. **d** Dominant model. **e** Recessive model. The squares and horizontal lines correspond to the study specific OR and 95% CI. The area of the squares reflects the weight. The diamond represents the summary OR and 95% CI. The fixed-effects model was used

In the analysis stratified by ethnicity, we found that rs1520220 was significantly associated with increased cancer susceptibility in Asian populations (Table 3 and Fig. 2, allele model OR = 1.10, 95%Cl = 1.00–1.21, *p* = 0.040; homozygote model OR = 1.22, 95%Cl = 1.01–1.47, *p* = 0.040; dominant model OR = 1.19, 95%Cl = 1.01–1.39, *p* = 0.033). Thus, no significant association was detected in Caucasian populations (Table 3 and Fig. 2).

In the analysis stratified by cancer type, the results show that rs1520220 was not associated with susceptibility to breast cancer (Table 3). Also, the results synthesized from studies that scored no less than 12 did not exhibited any differences from the results of the overall analysis (Table 3).

### Sensitivity analysis

**Fig. 3:**
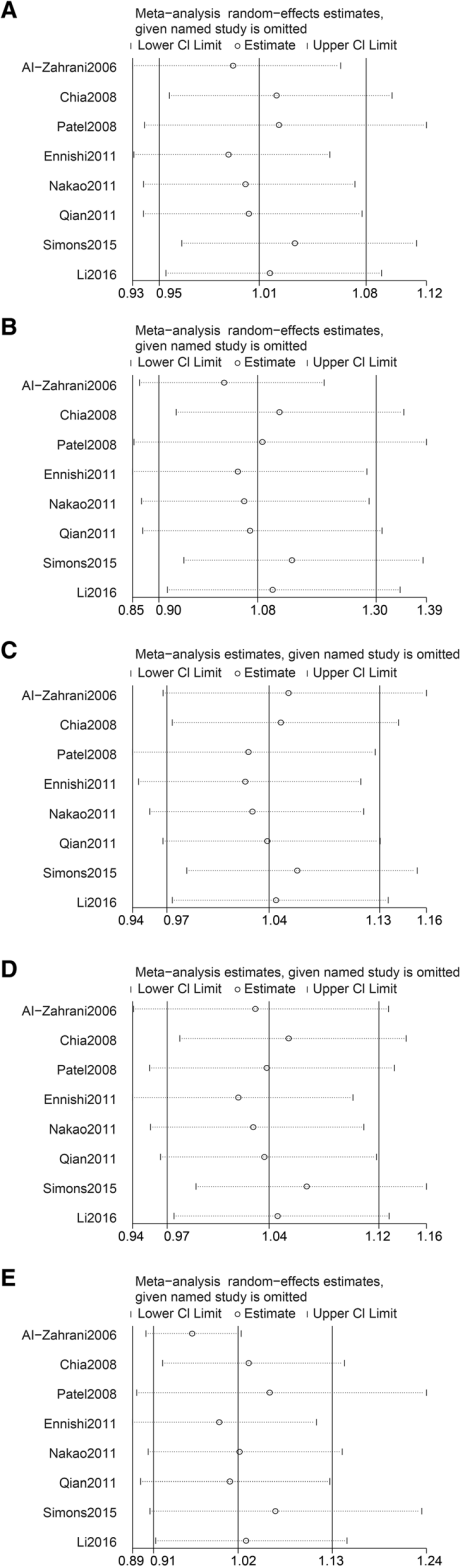
Sensitivity analyses between *IGF1* rs1520220 polymorphism and cancer susceptibility. **a** Allele model. **b** Homozygous model. **c** Heterozygous model. **d** Dominant model. **e** Recessive model. **a**, **b**, and **e**, the random-effects model was used. For **c** and **d**, the fixed-effects model was used

We performed sensitive analysis and found there was not an individual study that affected the results of the overall analysis (Fig. 3 and Additional file 1: Table S2), indicating that in this meta-analysis, our results are relatively stable.

### Publication bias

**Fig. 4:**
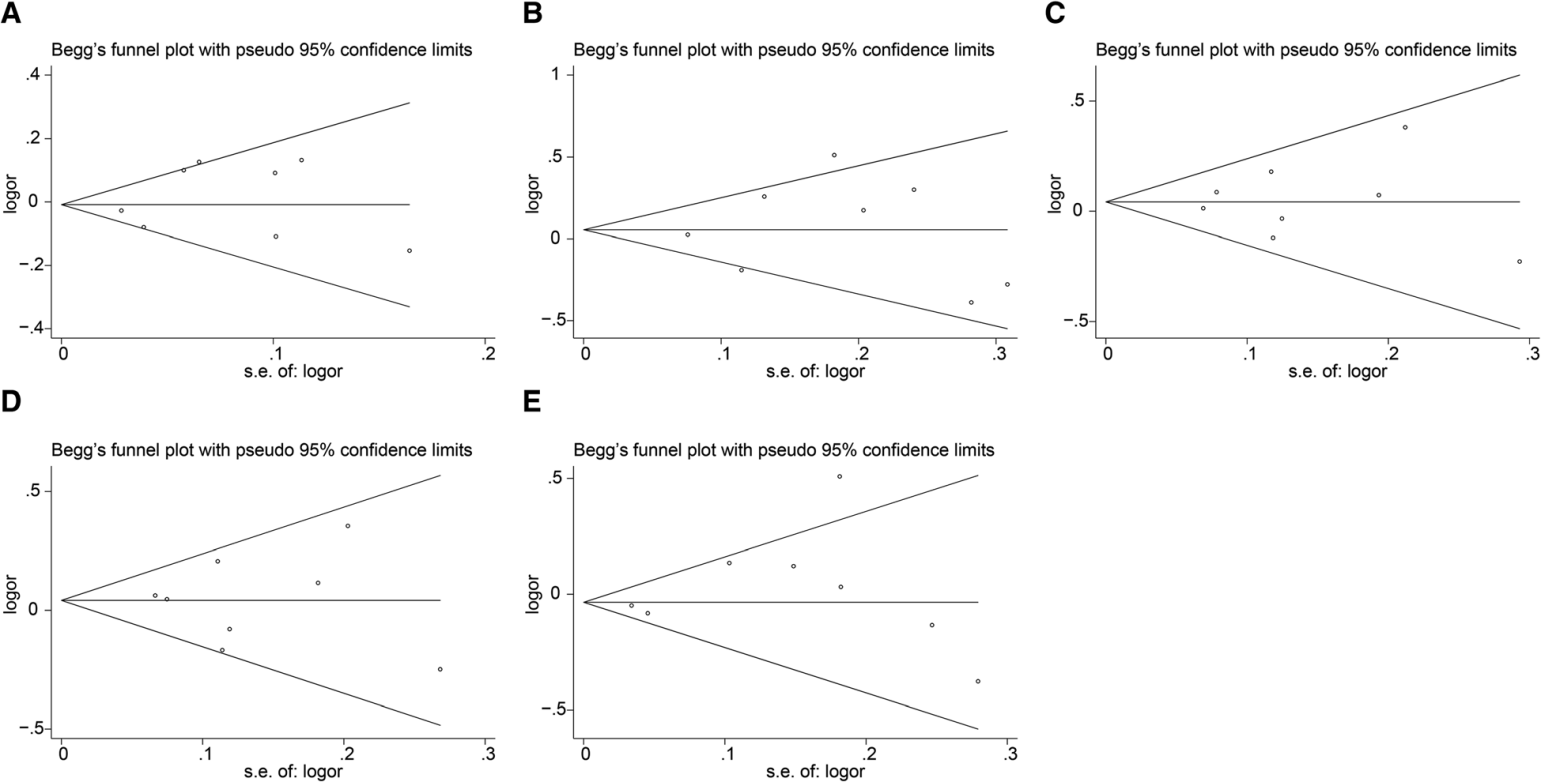
Funnel plot for publication bias test. **a** Allele model. **b** Homozygous model. **c** Heterozygous model. **d** Dominant model. **e** Recessive model

Begg’s test and Egger’s test were performed to assess the publication bias among the included studies. No publication bias was detected in the present meta-analysis (Fig. 4 and Table 4).

**Table 4:** Publication bias analysis

Genetic model	Egger’s test			Begg’s test
	*t*	95%Cl	*p*	*p*
C vs. G	0.85	− 1.731~3.568	0.429	0.902
CC vs. GG	0.23	− 3.080~3.724	0.824	0.902
GC vs. GG	0.14	− 2.255~2.537	0.890	1.000
CC + GC vs. GG	− 0.09	− 2.981–2.760	0.928	0.902
CC vs. GC + GG	1.11	− 1.099–2.912	0.311	0.902

## Discussion

Many SNPs have been reported to be associated with cancer susceptibility and thus may have the potentiality as biomarkers for clinical diagnosis [5–7]. Thus far, with the improvement of living standards in more and more developing countries, obesity and lifestyle-related cancers have been on the increase [33, 34]. IGF1 has been reported to relate to be associated with the cancer susceptibility, especially cancers caused by obesity, due to its important role in cell proliferation [35].

Several *IGF1* SNPs have been reported to be associated with cancer susceptibility [36–39]. These SNPs include rs1520220, rs6214, rs6220, rs35767, and rs5742612. Of these, rs6214 and rs6220 are located in the 3′-UTR region of the *IGF1* gene. It has been reported that rs6214 is associated with increased esophageal adenocarcinoma (EAC) and head and neck cancer (HNC) susceptibility in women [39] and that rs6220 is associated with increased prostate cancer susceptibility [40]. The rs35767 and rs5742612 SNPs are located in the promoter region of the *IGF1* gene. It has been reported that rs35767 is significantly associated with increased susceptibility of childhood acute lymphoblastic leukemia (ALL) [41] and that rs5742612 is associated with increased susceptibility to prostate cancer [42].

rs1520220 is an SNP that is located in the intron of the *IGF1* gene, and it has a minor allele frequency (MAF) about 10~ 40% in the populations included in the human 1000 Genomic Project phase 3 (Additional file 1: Table S3). We paid special attention to rs1520220 because it has been reported to be associated with plasma IGF1 levels in many studies and thus associated with cancer susceptibility [13, 17, 18].

In this meta-analysis, we systematically searched for literature on *IGF1* SNPs and cancer in three important databases (PubMed, Embase, and Web of Science). After removing duplicate documents, 2086 related articles were initially obtained, which ensured the maximum possible recall rate. Through meta-analysis, we found that rs1520220 was not related to cancer susceptibility in the overall analysis based on the present epidemiology studies. Thus, in the analysis stratified by ethnicity, we revealed that rs1520220 increased cancer susceptibility in Asian populations.

The present studies regarding the effect of *IGF1* rs1520220 polymorphism on serum IGF1 are inconsistent [13, 18]. In brief, rs1520220 may influence circulating IGF1 expression by altering the secondary structure of the RNA or DNA [16], and this effect may be enhanced by dietary factors [43]. Therefore, we infer that rs1520220 affects cancer susceptibility in Asians but not other populations due to the combined effects of genetic and environmental factors. The mechanism via which rs1520220 affects serum levels must be investigated in the future.

Our meta-analysis has several limitations. Firstly, we found that rs1520220 increased cancer susceptibility in Asians. The molecular mechanism via which the rs1520220 C allele increases plasma IGF1 levels and thus cancer risk remains unclear. Secondly, we did not consider potential external factors, such as gender, age, diet, and tobacco and alcohol intake habits or gene-gene interactions. Thirdly, the number of studies included in the meta-analysis is limited. We only included studies written in English, and important-related studies in other languages may have been overlooked.

## Conclusion

The present meta-analysis showed that *IGF1* rs1520220 is not significantly associated with overall cancer susceptibility. However, we did find that rs1520220 significantly increased cancer susceptibility in Asian populations. We also suggest that rs1520220 was not associated with susceptibility to breast cancer. There is a need for additional well-designed epidemiology and molecular biology studies to verify these conclusions and provide new insights into the role of SNPs in the etiology of cancer.

## Additional file

Additional file 1: **Table S1.** Quality score assessment. **Table S2.** Sensitivity analyses for *IGF1* rs1520220 polymorphism and cancer susceptibility. **Table S3.** MAFs of *IGF1* rs1520220 polymorphism in the populations from the 1000 Genomes Project Phase 3. (DOCX 30 kb)

## Abbreviations

3′-UTR: three prime untranslated region
ALL: Acute lymphoblastic leukemia
CI: Confidence interval
EAC: Esophageal adenocarcinoma
HB: Hospital-based
HNC: Head and neck cancer
HWE: Hardy-Weinberg equilibrium
IGF1: Insulin-like growth factor 1
MAF: Minor allele frequency
OR: Odds ratio
PB: Population-based
SNP: Single nucleotide polymorphism
TGCT: Testicular germ cell tumors
