# Supplementary material for: The association between IGF1 gene rs1520220 polymorphism and cancer susceptibility: a meta-analysis based on 12,884 cases and 58,304 controls
Source: Environ Health Prev Med. 2018 Aug 16;23:38. doi: 10.1186/s12199-018-0727-y (PMC6094919; doi:10.1186/s12199-018-0727-y)
Supplement: Supplementary file 1 — Table S1. Quality score assessment. Table S2. Sensitivity analyses for IGF1 rs1520220 polymorphism and cancer susceptibility. Table S3. MAFs of IGF1 rs1520220 polymorphism in the populations from the 1000 Genomes Project Phase 3. (DOCX 30 kb) [file 12199_2018_727_MOESM1_ESM.docx]

**Scale for quality assessment criterion (26)**

|  | Criterion | Score |
| --- | --- | --- |
| **A** | **Source of cases** |  |
|  | Selected from population or cancer registry | 3 |
|  | Selected from hospital | 2 |
|  | Selected from pathology archives, but without description | 1 |
|  | Not described | 0 |
| **B** | **Source of controls** |  |
|  | Population-based | 3 |
|  | Blood donors or volunteers | 2 |
|  | Hospital-based (cancer-free patients) | 1 |
|  | Not described | 0 |
| **C** | **Specimens used for determining genotypes** |  |
|  | White blood cells or normal tissues | 3 |
|  | Not mentioned | 2 |
|  | Tumor tissues or exfoliated cells of tissue | 0 |
| **D** | **Hardy–Weinberg equilibrium in controls** |  |
|  | Hardy–Weinberg equilibrium | 3 |
|  | Hardy–Weinberg disequilibrium | 0 |
| **E** | **Total sample size** |  |
|  | ≥1,000 | 3 |
|  | ≥500 and <1,000 | 2 |
|  | ≥200 and <500 | 1 |
|  | <200 | 0 |

**Table S1: Quality score assessment**

|  | **A** | **B** | **C** | **D** | **E** | **Score** |
| --- | --- | --- | --- | --- | --- | --- |
| AI-Zahrani2006 | 3 | 3 | 3 | 3 | 3 | 15 |
| Chia2008 | 3 | 3 | 3 | 3 | 3 | 15 |
| Patel2008 | 3 | 3 | 3 | 0 | 3 | 12 |
| Ennishi2011 | 2 | 1 | 3 | 3 | 3 | 12 |
| Nakao2011 | 2 | 1 | 3 | 3 | 3 | 12 |
| Qian2011 | 2 | 1 | 3 | 3 | 2 | 11 |
| Simons2015 | 3 | 3 | 3 | 3 | 3 | 15 |
| Li2016 | 2 | 3 | 3 | 3 | 1 | 12 |

A-E represents the corresponding criterion in the table of scale for quality assessment criterion.

**Table S2: Sensitivity analyses for *IGF1* rs1520220 polymorphism and cancer susceptibility**

| **Comparison** | **Study omitted** | **Estimate** | **[95% Confident Interval]** | **Effect model** |
| --- | --- | --- | --- | --- |
| **C vs. G** | AI-Zahrani2006 | 0.995 | 0.928-1.067 | Random |
|  | Chia2008 | 1.025 | 0.953-1.102 |  |
|  | Patel2008 | 1.026 | 0.936-1.125 |  |
|  | Ennishi2011 | 0.992 | 0.929-1.060 |  |
|  | Nakao2011 | 1.004 | 0.935-1.077 |  |
|  | Qian2011 | 1.006 | 0.935-1.082 |  |
|  | Simons2015 | 1.037 | 0.961-1.118 |  |
|  | Li2016 | 1.020 | 0.950-1.095 |  |
|  | Combined | 1.013 | 0.946-1.084 |  |
| **CC vs. GG** | AI-Zahrani2006 | 1.018 | 0.862-1.202 | Random |
|  | Chia2008 | 1.120 | 0.930-1.349 |  |
|  | Patel2008 | 1.088 | 0.852-1.390 |  |
|  | Ennishi2011 | 1.043 | 0.850-1.218 |  |
|  | Nakao2011 | 1.055 | 0.866-1.285 |  |
|  | Qian2011 | 1.066 | 0.868-1.309 |  |
|  | Simons2015 | 1.143 | 0.944-1.384 |  |
|  | Li2016 | 1.107 | 0.914-1.342 |  |
|  | Combined | 1.080 | 0.898-1.298 |  |
| **GC vs. GG** | AI-Zahrani2006 | 1.058 | 0.962-1.162 | Fixed |
|  | Chia2008 | 1.052 | 0.969-1.141 |  |
|  | Patel2008 | 1.027 | 0.939-1.123 |  |
|  | Ennishi2011 | 1.025 | 0.944-1.112 |  |
|  | Nakao2011 | 1.030 | 0.952-1.115 |  |
|  | Qian2011 | 1.041 | 0.962-1.127 |  |
|  | Simons2015 | 1.064 | 0.980-1.155 |  |
|  | Li2016 | 1.048 | 0.969-1.133 |  |
|  | Combined | 1.043 | 0.965-1.127 |  |
| **CC+GC vs. GG** | AI-Zahrani2006 | 1.033 | 0.944-1.130 | Fixed |
|  | Chia2008 | 1.057 | 0.978-1.143 |  |
|  | Patel2008 | 1.041 | 0.956-1.134 |  |
|  | Ennishi2011 | 1.020 | 0.943-1.104 |  |
|  | Nakao2011 | 1.031 | 0.956-1.112 |  |
|  | Qian2011 | 1.039 | 0.964-1.121 |  |
|  | Simons2015 | 1.070 | 0.989-1.157 |  |
|  | Li2016 | 1.049 | 0.973-1.130 |  |
|  | Combined | 1.043 | 0.968-1.123 |  |
| **CC vs. GC+GG** | AI-Zahrani2006 | 0.960 | 0.904-1.020 | Random |
|  | Chia2008 | 1.029 | 0.924-1.145 |  |
|  | Patel2008 | 1.054 | 0.893-1.244 |  |
|  | Ennishi2011 | 0.993 | 0.888-1.111 |  |
|  | Nakao2011 | 1.018 | 0.907-1.142 |  |
|  | Qian2011 | 1.006 | 0.897-1.127 |  |
|  | Simons2015 | 1.061 | 0.909-1.239 |  |
|  | Li2016 | 1.025 | 0.916-1.148 |  |
|  | Combined | 1.016 | 0.913-1.130 |  |

**Table S3: MAFs of *IGF1* rs1520220 polymorphism in the populations from the 1000 Genomes Project Phase 3**

| Populations | MAF |
| --- | --- |
| ACB | 0.458 |
| ASW | 0.434 |
| BEB | 0.372 |
| CDX | 0.478 |
| CEU | 0.131 |
| CHB | 0.432 |
| CHS | 0.443 |
| CLM | 0.191 |
| ESN | 0.369 |
| FIN | 0.187 |
| GBR | 0.192 |
| GIH | 0.325 |
| GWD | 0.354 |
| IBS | 0.182 |
| ITU | 0.299 |
| JPT | 0.442 |
| KHV | 0.444 |
| LWK | 0.429 |
| MSL | 0.406 |
| MXL | 0.227 |
| PEL | 0.218 |
| PJL | 0.286 |
| PUR | 0.264 |
| STU | 0.299 |
| TSI | 0.126 |
| YRI | 0.333 |

MAFs: minor allele frequencies; ACB: African Carribbeans in Barbados; ASW: Americans of African Ancestry in SW USA; BEB: Bengali from Bangladesh; CDX: Chinese Dai in Xi -shuangbanna, China; CEU: Utah Residents with Northern and Western European Ancestry; CHB: Han Chinese in Beijing, China; CHS: Southern Han Chinese; CLM: Colombians from Medellin, Colombia; ESN: Esan in Nigeria; FIN: Finnish in Finland; GBR: British in England and Scotland; GIH: Gujarati Indian from Houston, Texas; GWD: Gambian in Western Division in the Gambia; IBS: Iberian Population in Spain; ITU: Indian Telugu from the UK; JPT: Japanese in Tokyo, Japan; KHV: Kinh in Ho Chi Minh City, Vietnam; LWK: Luhya in Webuye, Kenya; MSL: Mende in Sierra Leone; MXL: Mexican Ancestry in Los Angeles USA; PEL: Peruvians from Lima, Peru; PJL:  Punjabi from Lahore, Pakistan; PUR: Puerto Rican in Puerto Rico; STU: Sri Lankan Tamil from the UK; TSI: Toscani in Italia; YRI: Yoruba in Ibadan, Nigeria; N/A: MAF not available in these populations in 1000 Genomes.
